# Sex related biases for attending to object color versus object position are reflected in reaction time and accuracy

**DOI:** 10.1371/journal.pone.0210272

**Published:** 2019-01-09

**Authors:** Robert F. McGivern, Matthew Mosso, Adam Freudenberg, Robert J. Handa

**Affiliations:** 1 San Diego State University, Department of Psychology, San Diego, CA, United States of America; 2 Colorado State University, College of Veterinary Medicine and Biomedical Science, Fort Collins, CO, United States of America; Middlesex University, UNITED KINGDOM

## Abstract

Processing of visual features related to objects and space relations occurs within separate cortical streams that interact with selective attention. Such separation has implications for cognitive development because the perception of ‘what’ and ‘where’ provide a neural foundation for the development of aspects of higher cognition. Thus, a small attentional bias in early development for attending to one aspect over the other might influence subsequent higher cognitive processing in tasks involving object recognition and space relations. We examined 134 men and women for evidence of an inherent sex-related bias for attending to basic perceptual features related to object discrimination versus object position. Each stimulus consisted of a circle located in one of 9 positions within a surrounding frame. Circles were one of three shades of blue or red. These stimuli were used in a match-to-sample paradigm where participants were required to match circles on the basis of color or spatial position. The first stimulus appeared in the center of the screen for 400 msec and the matching stimulus subsequently appeared for 400 msec oriented 5 degrees to the right or left of center. The same stimuli were used to test the perception of color and position, with order of testing counterbalanced across participants. Results showed significantly longer reaction times in females compared with males, with better accuracy to discriminate color when that color was tested before position. Males showed better accuracy when object position was tested before color discrimination. A second experiment employed the same procedure, but enhanced selective attention by adding an endogenous cue that predicted the right or left location for the appearance of the matching stimulus. This manipulation greatly attenuated the sex differences in reaction time and accuracy compared to Experiment 1, suggesting that the sex-related attentional biases are strongly coupled to bottom-up processing. Overall, the sex related attentional biases toward processing object characteristics versus object position location suggest a differential manifestation of biased competition between the weighted systems of dorsal and ventral stream processing. Results are discussed with how a developmental bias in the processing objects versus space relations may contribute to adult cognitive sex differences in humans and animals.

## Introduction

Our perception of the visual world relies on functional streams that originate in the primary visual cortex (V1) and operate in parallel to process objects and space relations. The ventral stream extends into the inferior temporal cortex to give rise to a semantic representation of objects through hierarchical processing of feature detectors in V1 for color and form. Feature detectors in V1 for motion provide the basis for the perception of space relations within the dorsal stream, which extends into the superior and medial aspects of the parietal lobe [1–3]. Thus when we look at a at a simple stimulus, such as a small red circle within a square frame, the ventral stream contributes to the perception of ‘what’ is in the visual scene (color, circle, square) and the dorsal stream contributes to the perception of the spatial relations among the objects (e.g., circle is in the left corner of the frame).

Although the streams operate in parallel, interconnections occur between feature detectors in V1, as well as at several levels within their hierarchical processing [4]. As a result, ventral stream processing of objects includes a coarse recognition of space relations, while dorsal stream processing of space relations includes an independent representation of objects [5–7]. Different aspects of visual perception involve subsystems that reside within these streams. Among these are visuospatial processing of abstract and real motion from egocentric and allocentric perspectives, as well as object characteristics related to shape, affective qualities, faces, or conceptual categories [8–9]. In addition, processing within and between the streams is modulated by feed-forward, reciprocal connections with prefrontal attentional networks that can integrate or enhance the perception of different features. This combination of serial and parallel processing creates a cognitively flexible system that is dynamically responsive to ongoing behavioral goals and current environmental and sensory context [8,9].

This cognitive flexibility is reflected in bottom up and top down processing. Top down processing involves selective attention and its capacity to modulate bottom-up processing through feed-forward loops in order to focus on information from one stream or the other to meet ongoing conscious goals. For instance, looking for friend in a crowd highlights processing of the friend’s facial configuration, while also restricting perceptual attention to movement. Bottom up processing is unconscious and prioritizes incoming sensory stimuli for processing on the basis of prior experience. An example is the hyper-responsiveness of PTSD patients to innocuous stimuli, which arises from unconscious association with a previous traumatic event [10].

The development of the visual system, like other cortical regions, is influenced by physiological and experiential factors [11–13]. One of these is early exposure to androgens. Based on a review of cognitive sex differences, such exposure appears to modulate the organization of dorsal and ventral stream processing in a sex dependent manner [14]. Behavioral studies of adults show that males are more likely to attend to space relations between objects, while females are more likely to attend to the objects and their characteristics. Tasks that favor males include targeting skills such as throwing darts, tracking accuracy of moving objects, as well as mental rotation of objects. Females excel in tasks such as implicit and explicit recognition memory for objects and their location, verbal fluency related to naming objects beginning with a specific letter of the alphabet, and episodic and autobiographical memory. This differential pattern suggests a sex related dichotomy for attention to objects versus space relations, which is best exemplified by the differential strategy between males and females in navigating the environment. Men perform better than women in situations that favors a strategy that relies on cardinal directions (North, East, South, West), whereas women perform as well as men or better when landmarks are available for orientation.

The underlying pattern of better attention to objects in females and better attention to space relations, including movement, is also present in early development. As neonates, males exhibit a greater preference for attending to a moving mobile, in contrast to females who show a preference for a human face [15,16]. By 9 months of age, males track moving objects with significantly greater interest than females [17]. Sex differences in processing abstract movement are also present as early as 5 months of age, with males showing a greater capacity than females for mental rotation [18]. This pattern continues into early and middle childhood, where males continue to excel in spatial tasks involving mental transformation or rotation of objects [19,20]. A review of these types of established cognitive sex differences, where males excel on tasks related to space relations and females excel on tasks involving object recognition, indicates that differential processing of visual information arising from the dorsal and ventral streams may play a role in these sex differences [14]. However, whether or not these differences are associated with sex-related biases for attending to basic perceptual elements processed by one stream or the other has not been established.

Attention is a multilevel process that includes at least three cortical/subcortical systems related to alerting, orienting, and executive control processing [21]. These attentional systems form distinct but overlapping networks that collectively bring unconsciously processed information into awareness. Several types of visual attention are recognized, with each showing a neural activation pattern that is modulated by current context and goals [22]. Spatial attention is the overt or covert process that directs focus to a particular location. Feature based attention deploys covert resources toward specific aspects of a stimulus such as color or position. Object based attention coordinates the perceptual grouping of features into visual objects.

Feature based attention for form or motion activates neuronal populations in both ventral and dorsal stream pathways, but shows enhanced activation in the dorsal stream when the attended feature is motion or location, and greater activation in the ventral stream when it is form and color [23]. However, the activational pattern that emerges is not a simple reflection of the physical features that are present in the visual field, but rather one that arises from a biased competition among different aspects of the stimuli that contribute to higher cognition, such as specific object features or behavioral affordances that serve contextual goals and conform to visual context [24,25].

The biased competition model provides a framework for appreciating how individual differences in perception might contribute to different cognitive strategies in solving tasks, as well as further an understanding of the neural foundation of cognitive sex differences. Although studies of sex differences in basic aspects of selective attention in men and women are limited, the pattern of results broadly indicates greater involvement of top-down processing in female attention. Bayliss et al. [26] examined sex differences in selective attention for detecting the location of a target stimulus (the letter T or L) presented to the right or left of center. Prior to the appearance of the target stimulus appearance, a non-predictive face cue was briefly presented in the center of the screen, in which the eyes of the face gazed to the right or left. In spite of the fact that the eye gaze cue was random and had no predictive ability, females responded significantly faster than males when the eye gaze was consistent with the location position. No sex differences in reaction time were observed when the eye gaze cue was inconsistent, nor were sex differences observed when the task included a non-symbolic exogenous cuing of location. In a study that did employ a symbolic endogenous cue (arrow) that predicted the location of the appearance of aneutral target stimulus (asterisk) with 80% accuracy, Merrit et al. [27] found that reaction times to the valid cue were significantly longer in females compared to males.

Stoet [28] employed a more complex attentional task that measured the influence of compatible and incompatible flanker stimuli on target discrimination of men and women. Greater distractibility was observed in women based on both longer reaction times and poorer discrimination accuracy when the flanker was incompatible with the target. This pattern was also observed in a subsequent study using the Simon task to examine sex differences in spatial perceptual processing [29]. The participant was shown a rectangular box containing an arrow pointing to the right or left. On each trial, the box appears to the left or right side of the screen and the participant’s task is to indicate whether the arrow points right or left. Conflicting perceptual information arises when the arrow appears left of center with the arrow pointing right. Females showed significantly longer reaction times than males to make a correct decision under both compatible and incompatible conditions, but there were no sex difference in accuracy. Sex differences in reaction times were also observed in a different type of study where participants estimated the vector of a ball moving vertically toward a horizontal line. Females reaction times to estimate the intersect point were significantly longer than males [30].

Longer reaction times in women suggest broader attention to all objects in the visual environment, which reduces processing efficiency compared with men, but not necessarily accuracy. The differences can be viewed as cognitive styles; one biased toward first attending to objects and then to their relationship to the broader spatial environment, the other biased toward first attending to object location. These sex-related differences fit the electrophysiological pattern observed by Neuhaus et al. [31] showing fundamental differences in the visual evoked response of men and women performing the Attention Network Test. The N100 component, which is considered a reflection of perceptual discrimination, exhibited a second peak in occipital and prefrontal leads of females but not males, an effect that correlated with greater stimulus saliency in females. The second N100 peak has also been associated with greater cognitive effort and task difficulty [32]. Analyses estimating the source localization revealed significant increases in current density of the right extra-striate visual cortex and the right rostral prefrontal cortex in females compared to males, a pattern indicating greater top-down attention in females to salient stimuli. These sex differences occurred in the absence of any difference in accuracy[31]All visual stimuli inherently contain the perceptual elements processed by dorsal and ventral streams. Thus, for any given stimulus, an individual has the attentional capacity to focus on aspects processed by either stream in accordance with task demands. For simple recognition of perceptual features that have little relationship to higher cognition, experience, or education, one would expect reaction times for men and women to exhibit similar reaction times for processing the same stimulus. However, the studies reviewed above that show longer processing times in women for several simple perceptual tasks involving space relations suggest an inherent attentional bias in men and women related to processing of feature related information arising from dorsal and ventral streams.

The current studies were designed to examine whether such a low level bias could be directly demonstrated behaviorally. We reasoned that a sex-related perceptual bias would most likely be detected under relatively unstructured conditions of selective attention that have a minimal cognitive load. Toward the end, we employed a match-to-sample paradigm using simple stimuli in which a colored circle appeared within a square surround, thereby creating a stimulus in which competition between dorsal or ventral stream processing was resolved through selective attention. The circles were one of three shades of red or blue and were located in one of 9 positions within the square frame. The first stimulus appeared in the center of the screen and the matching stimulus subsequently appeared 5 degrees to the right of left of center. Each participant was exposed to two sets of trials, one that asked them to determine whether the two stimuli matched on color shade, the other whether they matched on location. The same stimuli were used in both conditions, with the order counterbalanced across participants. A second experiment used the same procedures and stimuli, but with the addition of an endogenous cue that predicted the right or left of center location for the appearance of the stimulus to match. The purpose of this addition was to reduce uncertainty and thereby enhance selective attention.

## Methods

### Participants

134 right-handed participants, 73 female (mean age = 19.28 years ± SD 0.80) and 61 males (mean age ± SD = 19.61 ± 2.59) were recruited from undergraduate psychology classes at San Diego State University. Participants provided information regarding age, handedness, sex, and visual acuity. Each was informed that the experiment was designed to measure color discrimination and object position recognition. All participants provided written informed consent and procedures were reviewed and approved by the Committee on Protection of Human Subjects at San Diego State University.

### Apparatus and stimuli

Stimulus presentation software was developed by Eugene Terehov (https://www.linkedin.com/in/eterehov) and the program was run on an Apple iMac with a 24” screen. Stimuli were created in Adobe Illustrator (version CS4) and saved in a Portable Network Graphics format. The application and the stimuli employed are available to be used for non-commercial research purposes at no cost by contacting the corresponding author.

**Fig 1:**
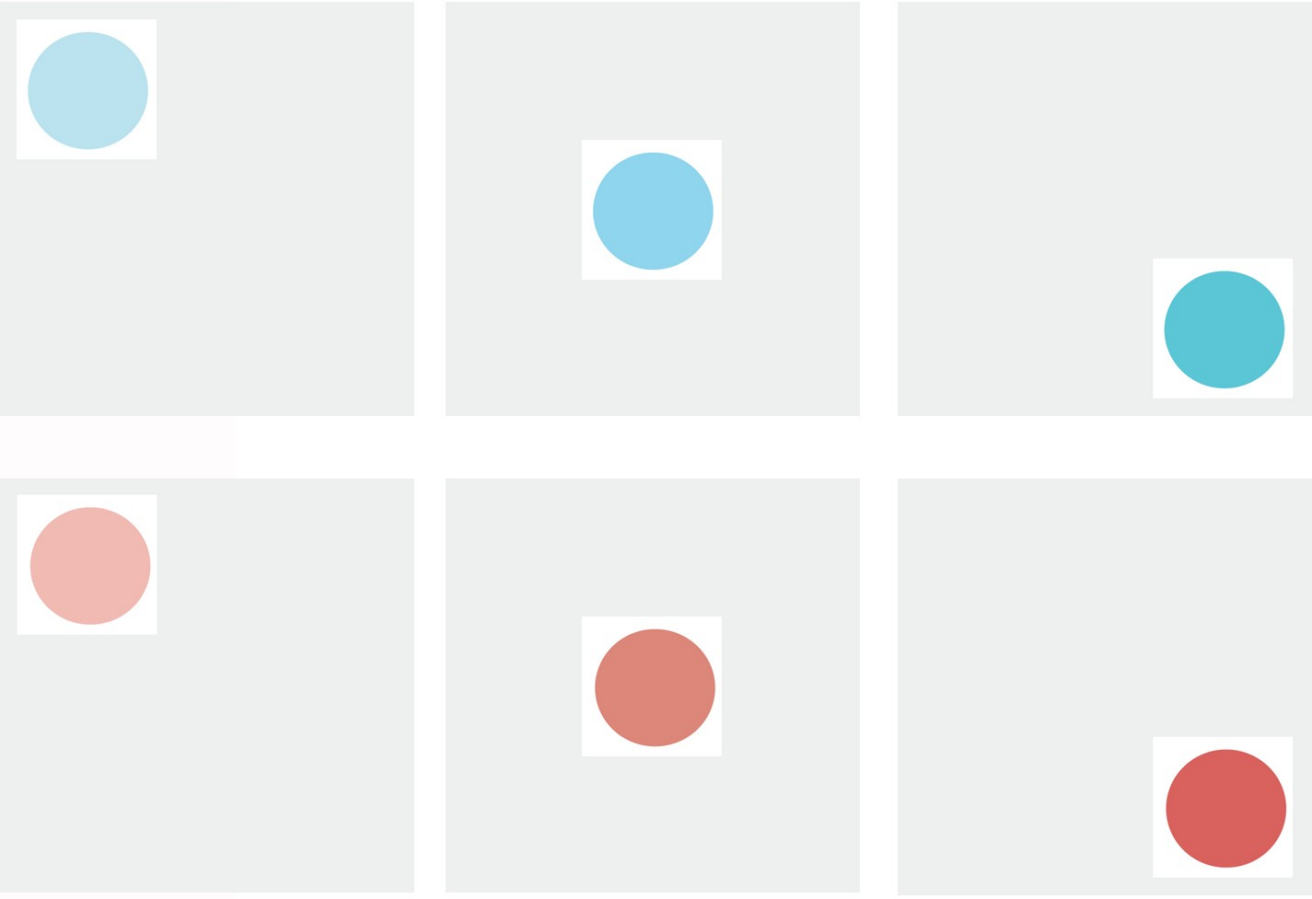
Stimuli examples. The three shades of the red and blue circles are shown set within 3 of the 9 available positions within the larger square background. RGB settings: Blue, 1) 185 224 234; 2) 145 211 234; 3) 95 197 214; Red, 1) 239 186 180; 2) 221 135 122; 3) 214 97 93.

The stimuli were designed to be used for two different sets of instructions to assess object position and color discrimination. Each stimulus was comprised of a 15mm colored circle appearing within one of nine equally spaced 18mm positions set within a 54mm square gray background. The circles were 15m diameter set within an 18mm white square to provide clear contrast. Three shades of blue or red were used for match on color. The RGB settings for the three shades of blue were: 1) 185 224 234; 2) 145 211 234; 3) 95 197 214. The shade settings for red were: 1) 239 186 180; 2) 221 135 122; 3) 214 97 93. the color of the circle to match could vary on color (blue or red) as well as color shade, with participants required to make a decision on whether two stimuli were an exact match. Examples of the stimuli and the 3 shades of red and blue employed are shown in Fig 1.

### Experiment 1. Uncued attention for perception of object color characteristics and object location

This experiment examined sex differences in the reaction time and/or accuracy for making a match-to-sample decision for discriminating colors and object position. Each was examined in separate sets of trials. Fifty-three participants were tested, with the data from 3 participants (2 male, 1 female) excluded due to mean accuracy scores or reaction times that were greater than 3 standard deviations from the same sex group. Fifty participants comprised the final sample for analysis (29 females).

### Procedure

Prior to testing selective attention, simple reaction time was measure in all participants. A plus sign appeared in the center of the screen for 750 milliseconds (msec), followed by a 15mm black circle presented to the right or left of center following a random interval of 350–750 msec. Participants pressed the ‘Q’ or ‘P’ key when they detected the stimulus. 20 trials were presented, with the mean of the last 15 trials used for analysis.

This was followed by eight match-to-sample practice trials using cartoon figures. The participant used the keyboard to indicate whether the stimuli were same or not by pressing the ‘Q’ key for a match and the ‘P’ key for a mismatch. Participants were then introduced to the formal experiment, which consisted of 80 trials. All colors and object positions were presented in a fixed, pseudorandom sequence. An automatic rest pause was inserted in the program every 20 trials, with the participant initiating the continuation of the experiment by pressing the space bar.

**Fig 2:**
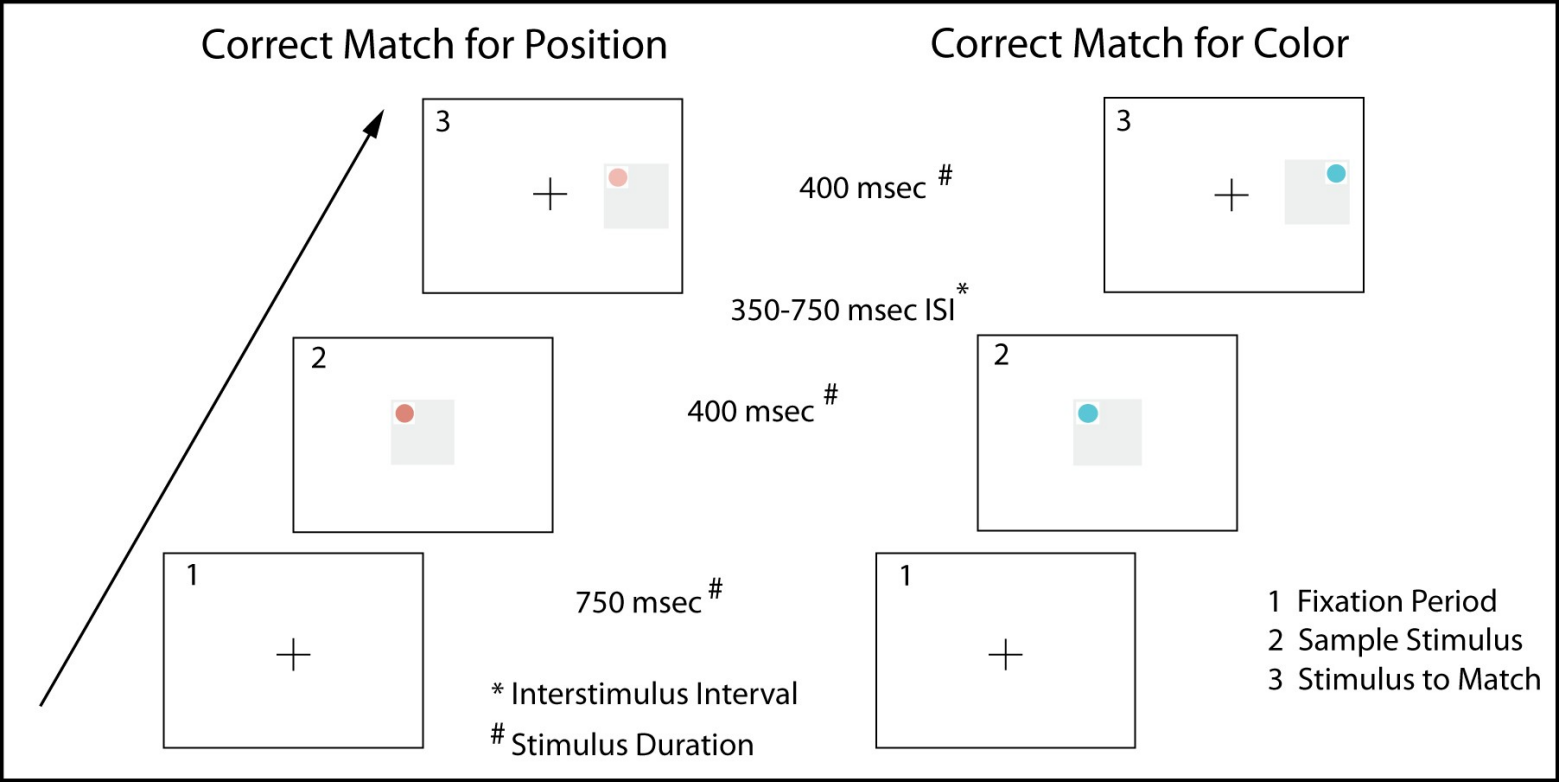
Flow chart for Experiment 1. The chart shows two possible correct answers that depend on the directions given. The sample trial on the left demonstrates matching the stimuli by position. The sample trial on the right demonstrates matching the stimuli by color. Panel 1 represents a fixation period where a plus sign was shown for 750 msec followed by a presentation of the stimulus for 400 msec in panel 2. There is a variable inter-stimulus of 350–750 msec followed by the target stimulus presentation for 400 msec, as represented in panel 3. Participants initiated a left hand response for a match and a right hand response for a mismatch.

At the beginning of each match-to-sample trial, a plus sign appeared for 750 msec to cue attention. After its offset, the first stimulus appeared in the same center location for a duration of 400msec. Following a random inter-stimulus interval (350–750 msec), the second stimulus was presented for 400msec in a pseudorandom order to the right or left of center at horizontal visual angle of 5°. The participant used the keyboard to indicate whether the stimuli matched by pressing the ‘Q’ key for a match and the ‘P’ key for a mismatch. Thus, for every trial there was a correct and incorrect answer. A flow chart for experiment 1 is shown in Fig 2.

Each participant was tested twice, once for matching on the object position and once for matching on the color. Both tests used the same procedure, including simple reaction time and practice trials. The two conditions were presented back-to-back, with total testing time that was approximately 20 minutes. The stimuli presentation order was the same in each condition, which meant that the perceptual basis for determining if the stimuli matched was determined only by the instructions. The testing order for perceptual matching was counterbalanced within sex.

Employing a match to sample paradigm was designed to activate top-down prefrontal attentional circuitry. However, the purpose of randomly offsetting the matching stimulus to the left or right was to also increase bottom-up processing, thus increasing the competitive processing balance involved in the decision. Our assumption was based on the fact that any inherent, sex related bias was likely to be dominated by bottom-up processing, and we expected this added uncertainty to help unmask such a bias.

We based the number of participants needed for experiment 1 on data from a preliminary study of 20 males and 23 females that used a longer stimulus presentation of 500 msec. Order was not systematically balanced across sex and more participants were tested in order 1 than order 2, so the results were collapsed across order. These preliminary results showed significantly greater accuracy for color discrimination and longer reaction times in females compared to males, and provided the basis for a power analysis of reaction time that estimated that 17 participants per sex were needed to meet or exceed a power value of 0.81 for color discrimination. We used this as the basis for the minimum number of each sex to test in experiment 1, but reduced the stimulus presentation time to 400 msec.

### Experiment 2. Attention for perception of object color characteristics and object location when a cue was empolyed to predicit for the right/left screen position for appearance of the matching stimulus

The procedures and methods were the same as in experiment 1 with the exception that a cue (arrow) was added at the start of the trial indicated the likelihood that the second stimulus would appear to the right or left of center following the first stimulus presented in the center. The arrow (pointing left or right of center) had an 80% probability of predicting subsequent location of the stimulus to match, but did not predict whether or not the stimulus matched the first. The purpose of employing the cue was to reduce attentional uncertainty and enhance selective attention to process aspects of object color or object location directly related to the matching tasks, thereby reducing potential inherent biases for attending to specific features.

The cue appeared for 400 msec after the offset of the plus sign. As in experiment 1, the first stimulus then appeared for 400 msec in the center position, followed by the stimulus to match appearing for 400 msec to the right or left of center. The practice trials in this condition also included the arrow. Each participant was tested for both location and object discrimination, with the order of condition counterbalanced within sex.

Eighty-three participants were tested for reaction time and/or accuracy using the same stimuli as in experiment 1, with data from 2 participants (2 female) excluded due to mean accuracy scores or reaction time that was greater than 3 standard deviations from the same sex group. Eighty-one participants comprised the final sample for analysis (44 females).

### Data analysis

The 80 match-to-sample trials were first cleaned of trials where reaction time exceeded 2000 msec. To further reduce variance, the remaining trials were subsequently cleaned of trials whose reaction times exceeded 2 standard deviations of the mean of the remaining trials. The average number of remaining trials for analysis was 76 (range: 74–78). No sex related pattern was found for reaction times that exceeded 2000 msec. The data for simple reaction time were used from the first perceptual condition tested in Experiment 1.

ANOVAs with repeated measures were conducted using BMDP Software. Reaction time was analyzed using a participant’s mean reaction time for each condition. Accuracy was analyzed using the percentage of correct answers based on the total trials included for analysis. In Experiment 1, trials were analyzed for reaction time and accuracy for identifying matching and non-matching pairs of stimuli. In Experiment 2, the data were sorted on accuracy for identifying matching and non-matching pairs under valid (arrow pointing to correct position) and invalid (arrow pointing to incorrect position) conditions. The limited number of invalid trials precluded a reliable statistical analysis of reaction time or accuracy.

## Results and discussion

### Experiment 1 results

**Fig 3:**
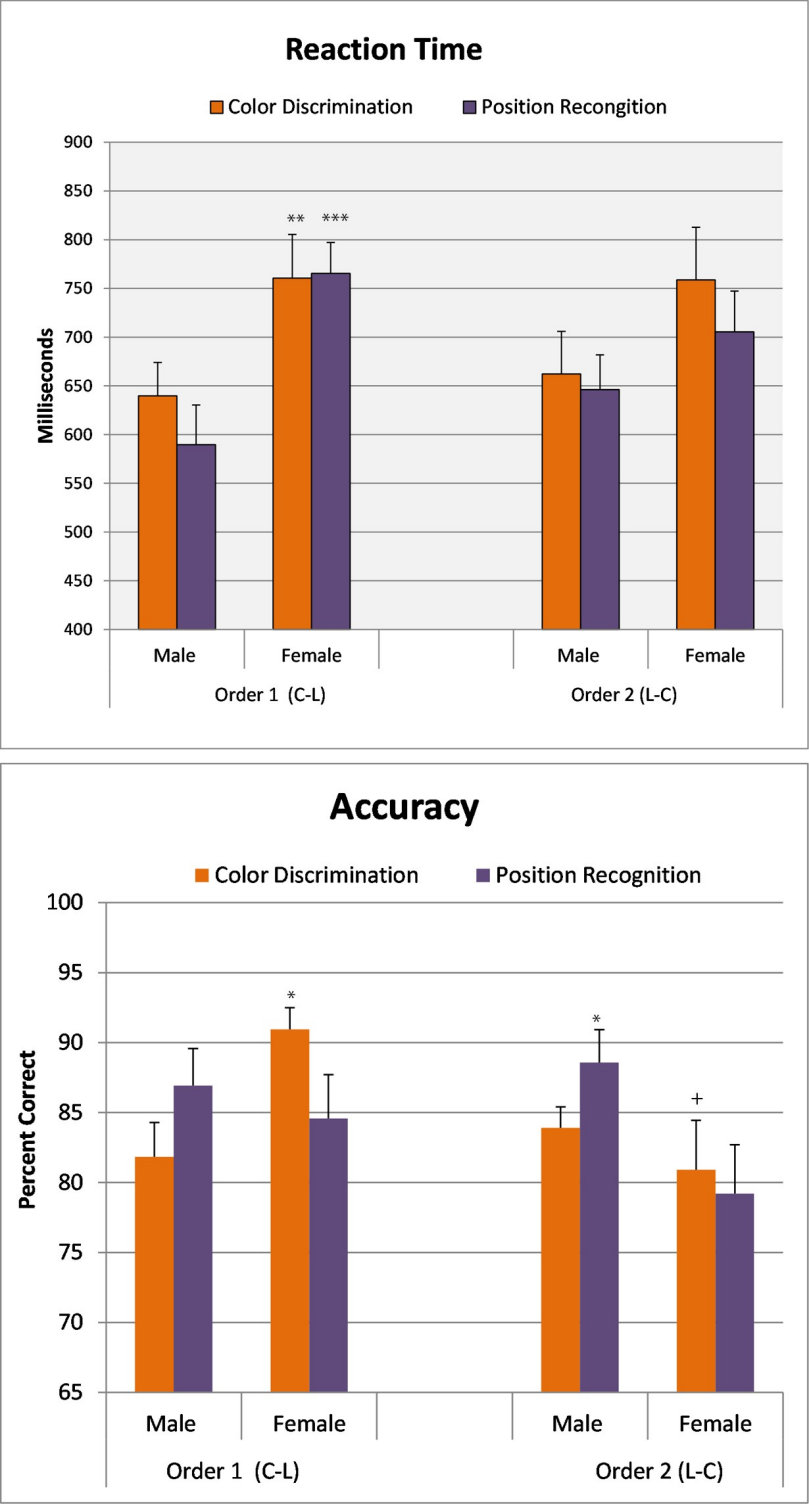
Reaction time and accuracy: Effect of intructional order for color and position matching. Data shown are the mean (± SEM). Order 1 tested for color first followed by testing for position. Order 2 tested for position first. Separate groups of males and females were tested in each order. **Top Panel:** Reaction time to make a correct matching decision (Yes/No). **p<0.01, ***p<0.001 compared to opposite sex in the same order. **Bottom Panel:** Percent of correct answers from same participants. *p<0.05 compared to opposite sex in same order. ^+^p<0.05 from same sex in opposite order.

Simple reaction time for detecting a stimulus to the right or left of center was similar in males and females (mean ± SD; Female, 328msec ± 0.025; Male, 322msec ± 0.033). Reaction times for correct answers and the percent correct were each analyzed using a 2(sex) X 2(order; Place/Color, Color/Place) X 2(condition; Color, Place) ANOVA with repeated measures over the condition factor. Significant main effects were found for Sex (F[1,46] = 8.47; p<0.006) and Perceptual Condition (F[1,46] = 4.00; p<0.05). As shown in Fig 3, the overall reaction time across condition and order in females was longer than males.

Accuracy in detecting object location and color discrimination showed significant sex differences related to the order of presentation (Sex x Order (F[1,46] = 4.46; p<0.04): Sex X Condition (F[1,46] = 5.79; p<0.025). As shown in Fig 3, when color discrimination was the first perceptual condition tested, female accuracy for color discrimination was significantly higher than males, as well as for females tested in the opposite testing order. Male accuracy was significantly higher than female for object location when object location was the first perceptual condition tested. A relationship of accuracy to reaction time was found only in males, where we found a significant correlation between longer reaction times and color discrimination accuracy (r = 0.51; p<0.02, 2-tail). This was not observed in females (r = 0.07) and no significant correlations were found in either sex between reaction time and position recognition.

### Experiment 2 results

**Fig 4:**
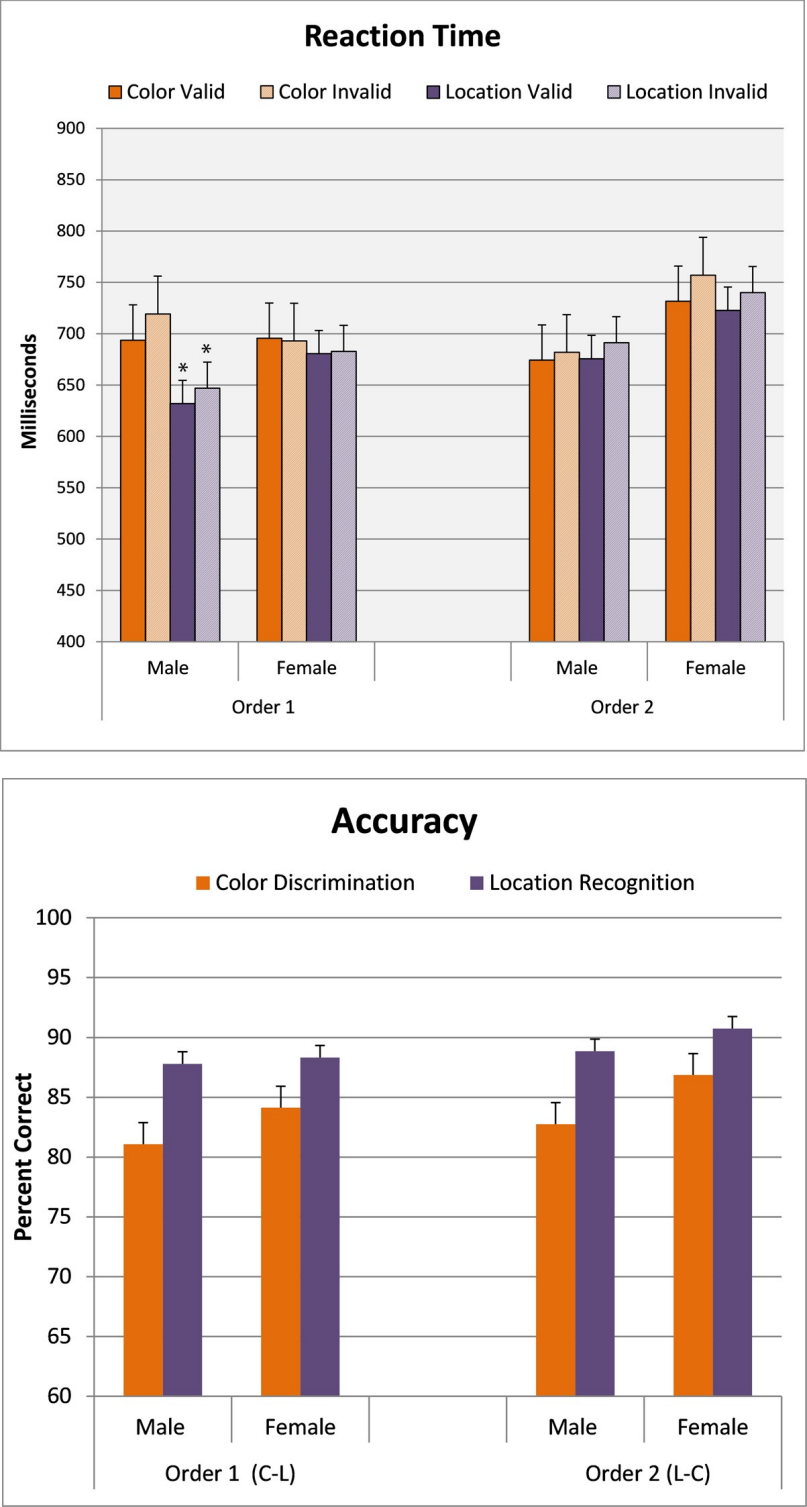
Influence of right/left of center for the appearnace of stimulus to match. Data shown are the mean (+ SEM) for reaction time and accuracy. **Top Panel:** reaction time to make a correct decision matching on color and position using a cue to predict the right/left of center appearance location of the stimulus to match. Cue was valid on 80% of the trials, but had no relationship to stimulus matching. *p<0.05 compared to opposite sex in same order. **Top Panel:** Reaction time to make a correct matching decision (Yes/No) for valid and invalid trials related to the endogenous positional cue. **Bottom Panel:** Percent of correct answers from same participants.

Reaction times for correct answers were analyzed using a 2(Sex) X 2(Order; place/color, color/place) X 2 (Condition; color, place) X 2 (Attention; valid, invalid) ANOVA with repeated measures in the Condition and Attention factors. Reaction times for both men and women significantly faster for location recognition versus color discrimination (Condition: F[1,77] = 4.32; p < .05). Overall reaction times in both sexes were also faster in the valid versus the invalid condition (Attention: F[1,77] = 13.23; p<0.0005). However, as shown in Fig 4 (top panel), this effect was influenced by the order of presentation (Attention X Sex X Order: F[1,77] = 4.32; p<0.05). Post hoc ANOVAs performed within sex showed that there was no significant effect of the valid cue on reaction time in females who were tested for color discrimination or in males who tested first for object location recognition. Accuracy for object and position recognition exceeded 80% and was similar for both men and women, with significantly better performance for object location recognition compared with color discrimination (F[1,77] = 27.04; p<0.0001). Results are depicted in the bottom panel of Fig 4.

## General discussion

The results of these studies reveal sex differences in both accuracy and reaction time to discriminate color or perceive object position, with women biased toward attending to color and men to attending to spatial position. The simple nature of the stimuli employed provides support for the hypothesized low level, sex-related bias in attending to information from one visual stream over the other. Because these differences were greatly attenuated when top down processing was enhanced by adding a predictive cue for right/left position of the matching stimulus, the sex-related biases appear to reflect differences in bottom-up processing of visual features.

In Experiment one, female reaction times were 15% longer than males when averaged across all testing conditions. Accuracy in Experiment one was also related to both order and sex. Sex differences were observed only when the first perceptual condition tested was in accordance with the hypothesized sex-related bias. When object position was the first perceptual condition tested, males were significantly more accurate than females, whereas when object color discrimination was the first condition tested, females were significantly more accurate than males. Correlation analyses found no significant relationship between accuracy and reaction time in females, but a positive correlation was observed in males between reaction time and object color discrimination. We found that when the first condition tested was object position males were significantly more accurate than females, whereas when the first condition tested was color discrimination females were significantly more accurate than males. This sex-related pattern in the relationship of accuracy to reaction time presents a complex picture, but overall points to processing and perceptual differences in the organization of attentional systems. Longer female reaction times to perceive object position, as well as color, are consistent with behavioral and imaging studies of cognitive sex differences showing greater top down processing in females for higher cognitive tasks involving objects and space relations, even in the absence of sex differences in performance [33–36].

An individual’s reaction time contains components of both top-down and bottom-up processing [37,38], but the proportion that each component contributes to the total is not fixed. Thus, longer reaction time for an individual may include more bottom-up processing. This is consistent with longer reaction times in females for implicit processing related to object recognition and object location memory [39,40]. The pattern implies differential neural organization for perceptual processing in males and females, which is indirectly supported by sex differences in structural connections of the brain [41]. In addition, cortical organization within each hemisphere is greater in males than between hemispheres, while the opposite pattern is seen in females. Perceptual connectivity in the male brain is also more oriented toward coordinated action, while that of females is more analytical and integrated across hemispheres. The greater female integration across hemispheres is consistent with imaging studies showing greater cortical activation when processing semantic information compared to males [42].

In Experiment two, the addition of a predictive cue for right/left appearance of the target stimulus eliminated the overall sex difference in reaction time observed in Experiment one. However, a sex difference remained in reaction time for position recognition that was related to testing order. When color recognition was the first perceptual condition tested, males were significantly faster than females to detect object position on valid and invalid trials. No other sex differences in reaction time on the basis or order or perceptual condition were observed.

The addition of the cue was expected to reduce reaction time on both sexes on valid trials. However, we found the predicted effect only when the first perceptual condition tested went against the sex-related bias. Thus, female reaction times for both color and position discrimination were significantly faster for a valid versus invalid cue only when position recognition was the first condition presented, while male reaction times for both perceptual conditions were significantly faster only when color discrimination was the first condition presented. We found no significant effect of the valid cue on reaction time when the participant was first tested with the condition that favored their bias (object position for men; color discrimination for women).

Several limitations of this study should be noted. First, we did not determine the stage of the menstrual cycle, a variable which has been shown to influence attention, reaction time, and spatial processing [43–45]. Second, we employed very basic perceptual stimuli in a match to sample paradigm in order to minimize cognitive load, distraction, and experience related to computer gaming or education. As a consequence, it is not clear how these might relate to other aspects of attention such as orienting, divided attention, or sustained attention. We also did not test participants for higher level cognitive tasks that show reliable sex differences, such as digit symbol, mental rotations, object location memory, or verbal fluency [46]. Thus, the degree of association between these low level attentional biases and higher cognitive skills will need to be addressed in future studies.

The influence of early attentional biases on the development of specific cognitive skills may be partly mediated by their effect on early cognitive and socio-emotional interests. Consistent sex differences in toy preferences are found in infants and children, with females showing a preference for the intrinsic properties of objects, such as movement, color, texture, or social associations (e.g. dolls), and males for objects that move or have a function (e.g, ball or truck) [47,48]. The appearance of this sex difference before 9 months of age, as well as findings showing that sex typed toy preferences in 4–5 year children have little relationship to conscious sexual stereotypes, indicate that these differences are not primarily related to socialization [49–51]. This is further supported by the existence of similar sex differences for toys in non-human primates [49,52].

The greater female attention to objects in infancy, especially faces, may play an important organizational role in subsequent socio-emotional development. Conversely, the early male interest in movement may enhance interest in object function in addition to spatial skills. In this way, organizational effects of hormones may induce an inherent attentional/perceptual bias toward processing information from one stream versus the other, thereby fostering greater interest in activities related to that processing. This in turn would stimulate greater neural development of the stimulated areas accompanied by sex differences in socio-emotional and cognitive behavior [14].

Facial perception is an example of this process. Faces have a basic static form that varies in the relative position or shape of features such as eyes, nose and mouth, as well as a dynamic process of changes among the features associated with emotional expressions. An important aspect of social interactions involves individual perception and attention to these features, with eye contact and joint attention serving as strong indicators of social attention and interaction. By one year of age, females show greater eye contact and joint attention than males when interacting with another individual [53–55]. Adult females also show an advantage over males in recognizing individual faces and emotional expressions [56,57]. It’s reasonable to assume that the sex difference in these perceptual skills play an important role in the greater empathy found in women compared to men [58,59], and may help to explain the greater reliance in women for using emotional salience as an important factor when making a moral judgment [60,61].

Finally, because dorsal and ventral streams are present in animals, cross-species studies of sex differences in these simple perceptual differences may serve as an evolutionary link to establishing a common neural substrate for cognitive sex differences in animals and humans [14]. This potential is perhaps best exemplified in a learning study in chickens conducted by Vallortigara [62]. Two groups of males and females were trained to discriminate between two adjoining squares based on color (red vs brown) or location (left vs right). No sex differences were found for discrimination learning based on condition (color or position). However, when the same animals were retrained to learn the opposite discrimination (reversal learning), males who first learned to discriminate color were significantly faster than females to learn the position cue, whereas females who first learned object position were significantly faster than males to learn to discriminate color. This cross species attentional bias, similar to the results of the present study, suggest that cognitive sex differences may arise in part from early physiological influences that modulate the behavioral salience of basic visual features.

## Supporting information

S1 File Supporting information. Data sets for Expariments 1 and 2.
(XLSX)
